# Death from Chloroform at Bellevue Hospital

**Published:** 1867-06

**Authors:** 


Death from Chloroform at Bellevue Hospital.—A death from chloroform took place recently at Bellevue Hospital on the occasion of the performance of a rhinoplastic operation by Professor Hamilton. The patient was a robust, middle-aged Irish woman, whose nose had been bitten off in an encounter with an negro. A preliminary operation had been made for the purpose of forming a flap for the new member, and evetything had progressed favorably. The second operation was intended to complete the formation of the nose, but before it could be commenced, the patient expired. It seems that chloroform had been first administered by two of the hospital internes with a view of bringing the patient rapidly under the influence of an anæsthetic, and an effort was afterwards made to employ ether, but insensibility not being satisfactorily induced, chloroform was again employed with the desired result, and the anæsthesia afterwards kept up by ether. As the operator was about to commence, the patient was noticed to grow suddenly pale, the pulse ceased at the wrist, and despite every exertion to resuscitate her by means, first, of artificial respiration, then of the galvanic battery, and afterwards by artificial breathing following laryngotomy, the patient expired. The materials were chemically pure, and the usual care was taken in their administration. Death took place as the result of paralysis of the heart.

